# Can Mediastinal Lymphadenopathy Signal Pericarditis, Pericardial Effusion, and Severe Disease in a COVID-19 Patient?

**DOI:** 10.7759/cureus.22160

**Published:** 2022-02-12

**Authors:** Nabeel Khan, Sarbagya Pandit

**Affiliations:** 1 Internal Medicine, Medical College of Wisconsin, Milwaukee, USA

**Keywords:** hilar lymphadenopathy, sar-cov 2 infection, covid 19, covid pneumonia, severe, pericardial effusion, mediastinal lymphadenopathy, pericarditis

## Abstract

Severe acute respiratory syndrome coronavirus 2 (SARS-COV-2) has created a global pandemic. As we try to understand the virus, we are learning that it can affect many organ systems. Most commonly coronavirus disease 2019 (COVID-19) virus affects the respiratory tract and the lungs impairing oxygen transport to the systemic circulation. Its effect on the cardiovascular system can be equally as devastating. Patients can develop pericarditis, myocarditis, and pericardial effusion that can at times lead to tamponade. Here we present an unusual case of a patient with COVID-19 pneumonia who presented with pericardial effusion along with enlarged mediastinal lymph nodes.

## Introduction

Coronavirus disease 2019 (COVID-19) infection generally presents with multiple health sequelae due to its pathophysiologic effect on different organ systems. Typically, COVID-19 presents with respiratory symptoms, and the appearance of ground-glass opacities on X-ray and CT is one of the most common findings [1]. According to reported studies, severe acute respiratory syndrome coronavirus 2 (SARS-CoV-2) infection is not limited to the respiratory system and other organs can be also affected [2]. Cardiovascular involvement has been reported in multiple studies and can involve myocardial injury, pericardial effusion, and arrhythmias [2]. We report a case of a patient with COVID-19 who had findings of pericardial effusion along with mediastinal lymphadenopathy.

## Case presentation

A 45-year-old African American female with a past medical history of asthma, hypertension, cervical cancer, and tobacco use presented to the hospital with the chief complaint of chest pain and discomfort the night prior to presentation. The patient had a positive polymerase chain reaction (PCR) test for COVID-19 three days before presentation. The patient’s 10-year-old daughter had COVID-19 which resolved, and the patient started to experience symptoms a few days after, which included dry cough and shortness of breath. She also reported one episode of hemoptysis. She had chest pain for a few days which worsened with deep breath and coughing and did not improve with leaning forward. She also had gastrointestinal (GI) symptoms including diarrhea and abdominal pain. Her medications included albuterol, amlodipine, hydrochlorothiazide (HCTZ), and omeprazole. Her initial vitals on presentation showed blood pressure (BP) 127/86 mmHg, heart rate (HR) 122/min, temperature 99 degrees Celsius, and saturation 99% on room air.

**Figure 1:**
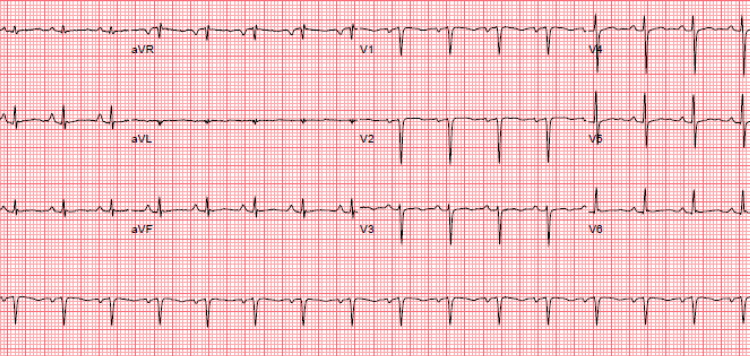
Initial EKG showed sinus tachycardia with no ST changes. EKG, electrocardiogram

Initial head and neck exam revealed midline trachea, no anterior or posterior lymphadenopathy. There was no jugular vein distension (JVD). She was tachycardic with a regular rhythm. There were no murmur, gallop, or rubs noted. A respiratory exam noted tachypnea with shallow breaths and bilateral crackles. Pulses were equal bilaterally and there was no edema noted on extremities. Initial electrocardiography (EKG) showed sinus tachycardia with no ST changes (Figure 1).

Hematology labs showed white blood cell (WBC) count of 5.7 x 103/µL (normal rage 3.9-11.2 x 103/µL) with segmented neutrophils of 44% (normal range 43%-74%) and 44% lymphocytes (normal range 17%-46%), and hemoglobin (Hb) 16.4 g/dL (normal range 11.3-15.1 g/dL). Coagulation study revealed elevated D-dimer of 0.67 mg/L (normal <0.5 mg/L). 

**Figure 2:**
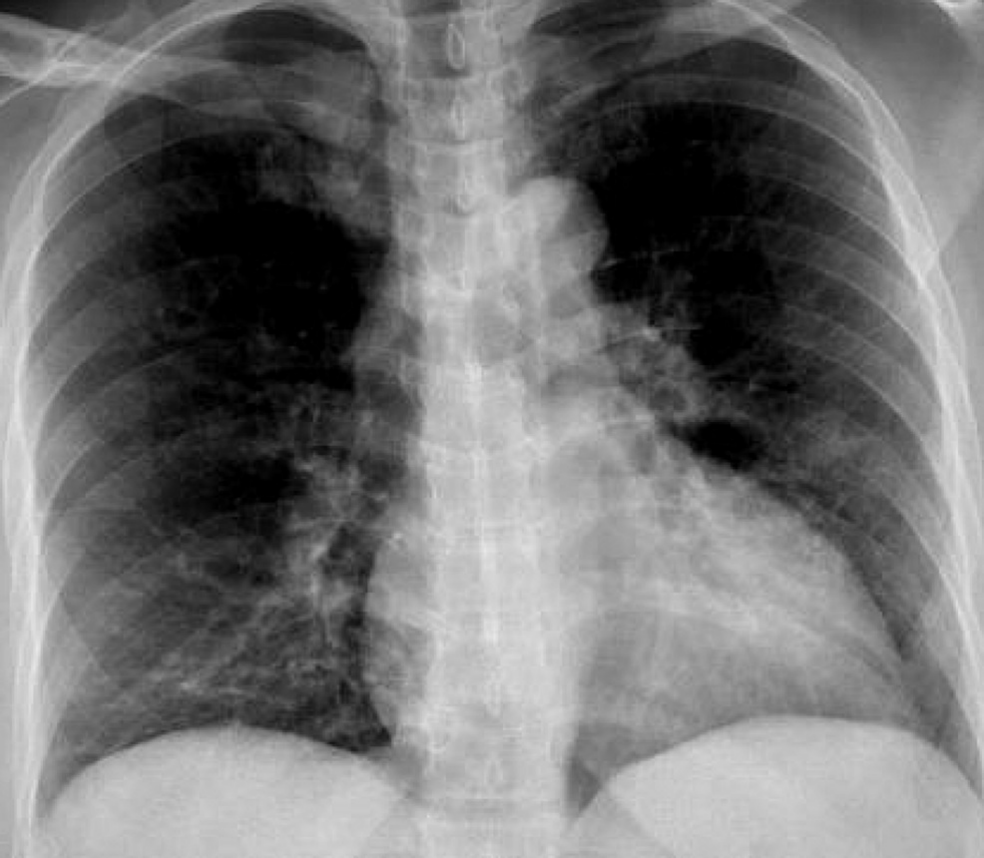
Chest X-ray showing subtle alveolar opacities in left midlung, linear opacities at the bases.

The complete metabolic panel was in normal range except for low potassium at 2.9 mmol/L (normal range 3.4-5.1 mmol/L), slightly increased alkaline phosphatase at 105 u/L (normal range 35-104 u/L), and aspartate aminotransferase (AST) 107 u/L (normal range 11-33 u/L). Lactic acid was 2.3 mmol/L (normal range 0.5-5 mmol/L). Initial high sensitivity troponin was 45 ng/L (normal range <10 ng/L) with 2 h repeat at 41 ng/L and delta of 4. Brain natriuretic peptide (BNP) was elevated at 687 pg/mL. Chest X-ray showed subtle alveolar opacities in the left midlung, linear opacities at the bases (Figure 2).

**Figure 3:**
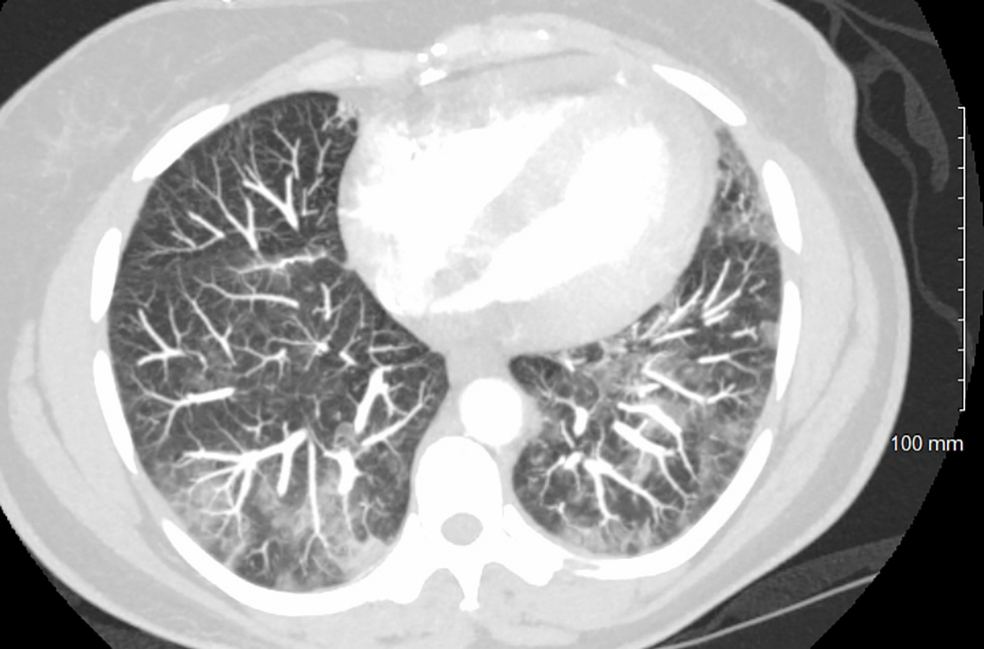
CT angiogram was negative for pulmonary embolism, however, did show multifocal airspace opacities consistent with COVID-19, small to moderate sized pericardial effusion.

**Figure 4:**
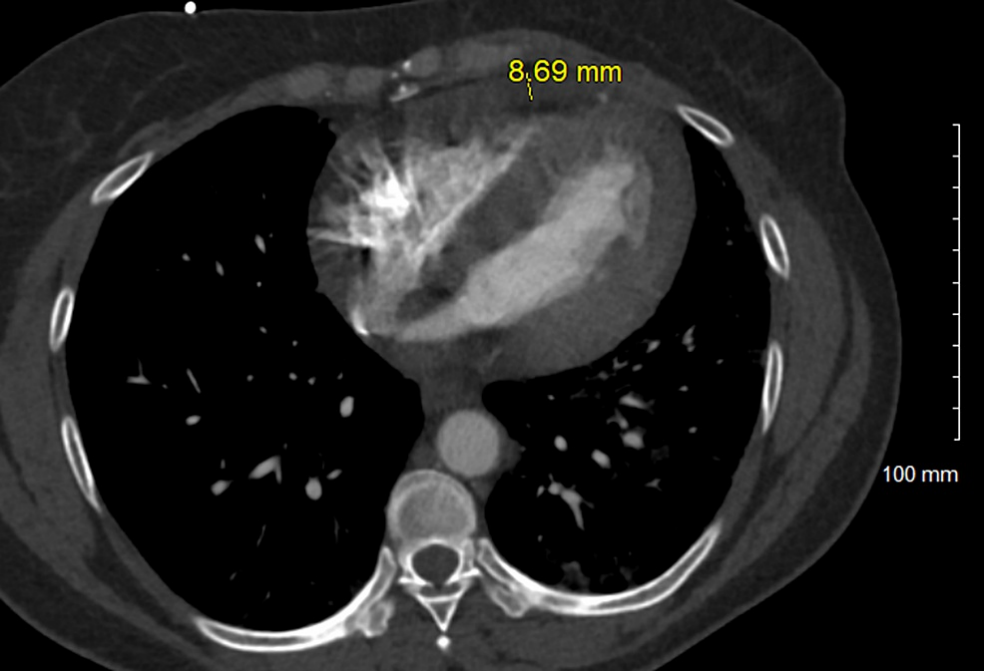
Moderate sized pericardial effusion.

**Figure 5:**
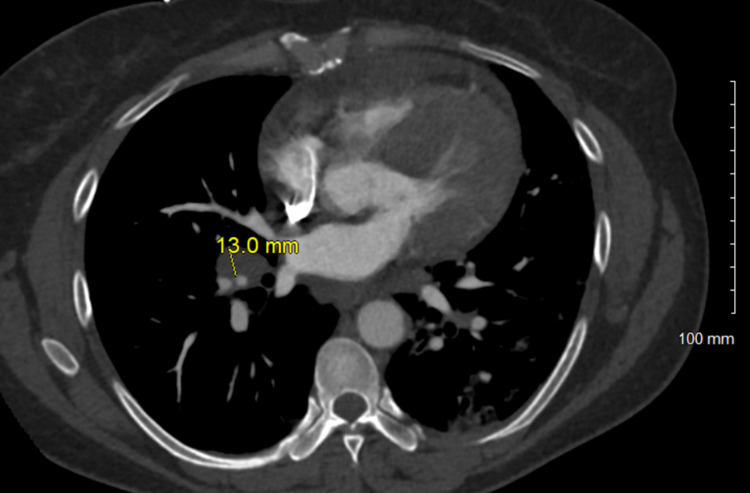
Enlarged right hilar lymph nodes, 13.0 mm.

**Figure 6:**
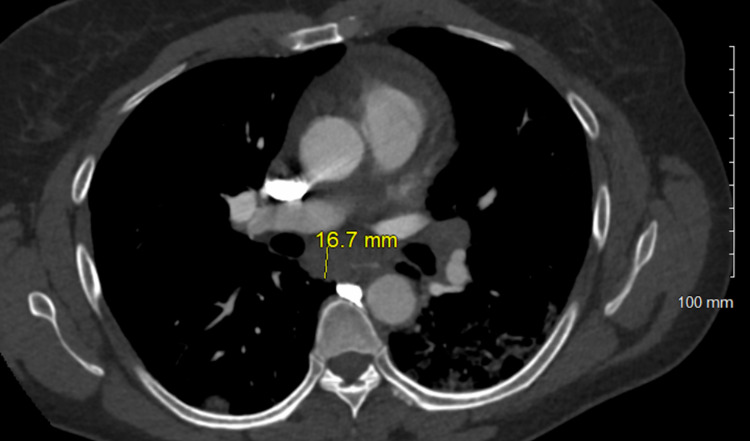
Enlarged mediastinal lymph node, 16.7 mm.

**Figure 7:**
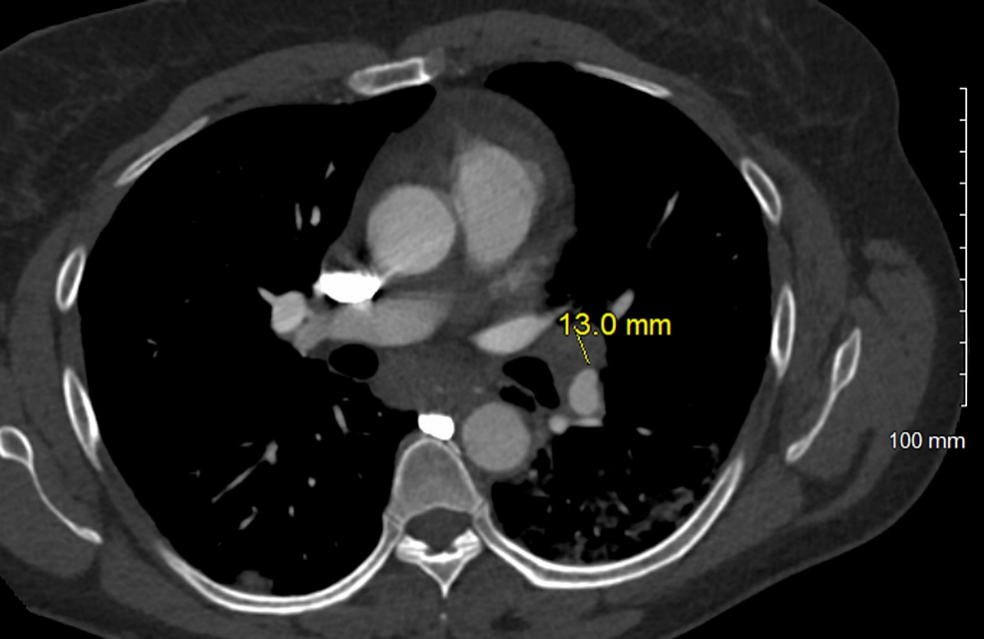
Enlarged left hilar lymph node, 13.0 mm.

Due to dyspnea, tachycardia, and elevated D-dimer, CT angiogram was ordered which was negative for pulmonary embolism (PE), however, did show multifocal airspace opacities consistent with COVID-19 (Figure 3), small to moderate sized pericardial effusion (Figure 4) suggestive of underlying pericarditis and enlarged mediastinal and hilar lymph nodes (Figures 5-7). A transthoracic echocardiogram (TTE) was ordered which showed an ejection fraction (EF) of 66% with concentric left ventricular hypertrophy, pericardial effusion adjacent to the right ventricle free wall. There was significant respiratory variation in the mitral inflow which may be consistent with early tamponade physiology (may also be seen in obesity or lung disease).

It was believed that the likely cause of effusion was COVID-19 as the patient was symptomatic and had signs of pneumonia. She was not tested for other causes of pericardial effusion such as lupus or human immunodeficiency virus (HIV). The cardiologist was consulted who recommended conservative treatment with high dose nonsteroidal anti-inflammatory drugs (NSAIDS) for seven days and colchicine for three months. Due to hypoxia, the patient received dexamethasone, remdesivir and two units of convalescent plasma and developed transfusion-associated circulatory overload. BNP increased to 984 pg/mL. Due to worsening oxygen requirements, she was transferred to the intensive care unit (ICU). Symptoms improved with diuresis and she was transferred back to the floor. Her oxygen requirements improved to room air and she was discharged with a few remaining days of dexamethasone and three months of colchicine along with cardiology follow-up in two to three weeks.

## Discussion

The causes of pericarditis are numerous as any process that inflames, injures, or reduces lymphatic drainage of the pericardium can result in an effusion [3]. Idiopathic pericarditis, usually presumed to be post-viral is the most common cause of an inflammation-related pericardial effusion in the United States and Western Europe [3]. Tuberculosis is the most common cause in the developing world [3]. Pericarditis is a rare finding seen in patients with novel SARS-CoV2. A recent metanalysis showed the occurrence of pericardial effusion in 4.55% of patients with COVID-19 [1]. There have been several reports of pericarditis with large effusion and tamponade requiring therapeutic pericardiocentesis. Analysis of fluids in all patients was exudative with high lactate dehydrogenase (LDH) and albumin and serum to fluid LDH ratio >0.6 [4]. Myopericarditis is an extension of inflammation to the myocardium with elevated troponin but without left ventricular (LV) systolic dysfunction.

Mediastinal lymphadenopathy is found in severely ill COVID-19 patients [2]. Meta-analysis revealed 3.38% of patients had a presentation of lymphadenopathy on CT scan [1]. The size of the lymph nodes is a strong predictor of etiology with 15 mm or less more likely reactive and larger than 25 mm likely pathologic due to malignancy or sarcoidosis [5]. Since cardiac and pulmonary lymphatic drainage is to the mediastinal lymph nodes, it would be reasonable to assume that these lymph nodes enlargement will be more likely when both organs are involved or if there is a severe disease. Furthermore, enlarged mediastinal lymph nodes are independently associated with increased mortality in COVID-19 infection [6]. 

Angiotensin-converting enzyme 1 (ACE1) as a target receptor for SARS-CoV-2 is significantly expressed in the heart [2]. This transmembrane aminopeptidase involved in the development of hypertension and heart function performs an extremely critical role in the cardiovascular system [2]. The exact incidence of cardiac involvement in COVID-19 is unknown. Myocardial injury was observed in 7%-17% of patients [4]. Mortality rate and N-terminal pro-B type natriuretic peptide (NT-proBNP) levels are significantly higher in patients with myocardial injury [2].

In the case report we presented, myopericarditis was present along with enlarged mediastinal lymph nodes. There have been reports of each one present individually but not many where both are present. We believe that there might be a link between the presence of mediastinal lymphadenopathy and the finding of pericarditis. Further evaluation might be needed to see if there is a correlation between the two.

## Conclusions

Various forms of cardiac involvement have been reported due to COVID-19 virus infection and include myocardial infarction, cardiogenic shock, decompensated heart failure, and pericardial effusion. Patients with severe disease can present with mediastinal lymphadenopathy. Lymphadenopathy may be related to pericardial effusion or myopericarditis. Patients can be asymptomatic or have chest pain. Pericardial effusion is treated based on size and its effect on cardiac function. It is medically treated with NSAIDs and colchicine.
